# The metabolic environment within solid tumors drives a complex crosstalk between macrophages and NK cells

**DOI:** 10.3389/fimmu.2026.1726590

**Published:** 2026-03-20

**Authors:** Didem Cakirsoy Akyoney, Joel Nordin, Samir El Andaloussi, Evren Alici

**Affiliations:** 1 Department of Medicine, Karolinska Institutet, Huddinge, Sweden; 2 Department of Laboratory Medicine, Karolinska Institutet, Stockholm, Sweden

**Keywords:** anti-tumor immunity, crosstalk, macrophage, metabolic competition, natural killer cells (NK cells), solid tumor, tumor associate macrophages (TAM)

## Abstract

The tumor microenvironment (TME) exerts significant metabolic limitations that influence the activity of invading immune cells. Among them, macrophages and natural killer (NK) cells are essential for coordinating anti-tumor immunity; however, the metabolic conditions of solid tumors have a significant impact on their functional states. Emerging evidence indicates that metabolic competition and nutrition availability regulate the dynamic interactions between these two innate immune populations, eventually influencing immune activation, suppression, and tumor growth. In this review, we discuss how key metabolic factors, including glucose depletion, lipid metabolism, hypoxia, and lactate accumulation, reshape NK cell activity and macrophage polarization in the TME. We emphasize how cytokine signaling and spatial organization within tumors influence NK-macrophage interactions, resulting in either synergistic anti-tumor responses or immunosuppressive networks. Finally, we explore novel therapeutic approaches designed to target metabolic pathways to restore NK cell function and reprogram macrophages toward pro-inflammatory phenotypes. Understanding the metabolic regulation of NK-macrophage interactions could provide new opportunities to improve immunotherapy efficacy in solid tumors.

## Introduction

Tumor cells use various immunosuppressive mechanisms to escape immune-mediated killing, which allows them to persist and thrive throughout the anti-tumor immune response. This "immune escape" phenomenon occurs when tumor cells display strategies to suppress the immune system (1). Cancer cells can evade immune monitoring by hindering antigen presentation, using negative regulation, and recruiting immunosuppressive cell populations, even when nonspecific immunity is naturally successful (2). As a result, immune cell effector activities are weakened, and anti-tumor immune responses are ineffective (3). With the idea of strengthening immune cells' anti-cancer properties to stop cancer cells from escaping the immune system, immunotherapy was developed in response to this difficulty (4).

Immunotherapies against hematological malignancies have demonstrated higher success than solid tumors (5). However, according to US 2020 data, solid tumor cases are ten times higher than hematological cancers (6). From this perspective, solid tumor immunotherapy studies are of great importance. There is an obvious need for successful immunotherapies in solid tumors compared to hematological malignancies (7). Within this context, natural killer (NK) cells and macrophages are one of the key players in the anti-tumor immune response and play a central role in shaping immune activity within the tumor microenvironment.

## Solid tumors

**Figure 1:**
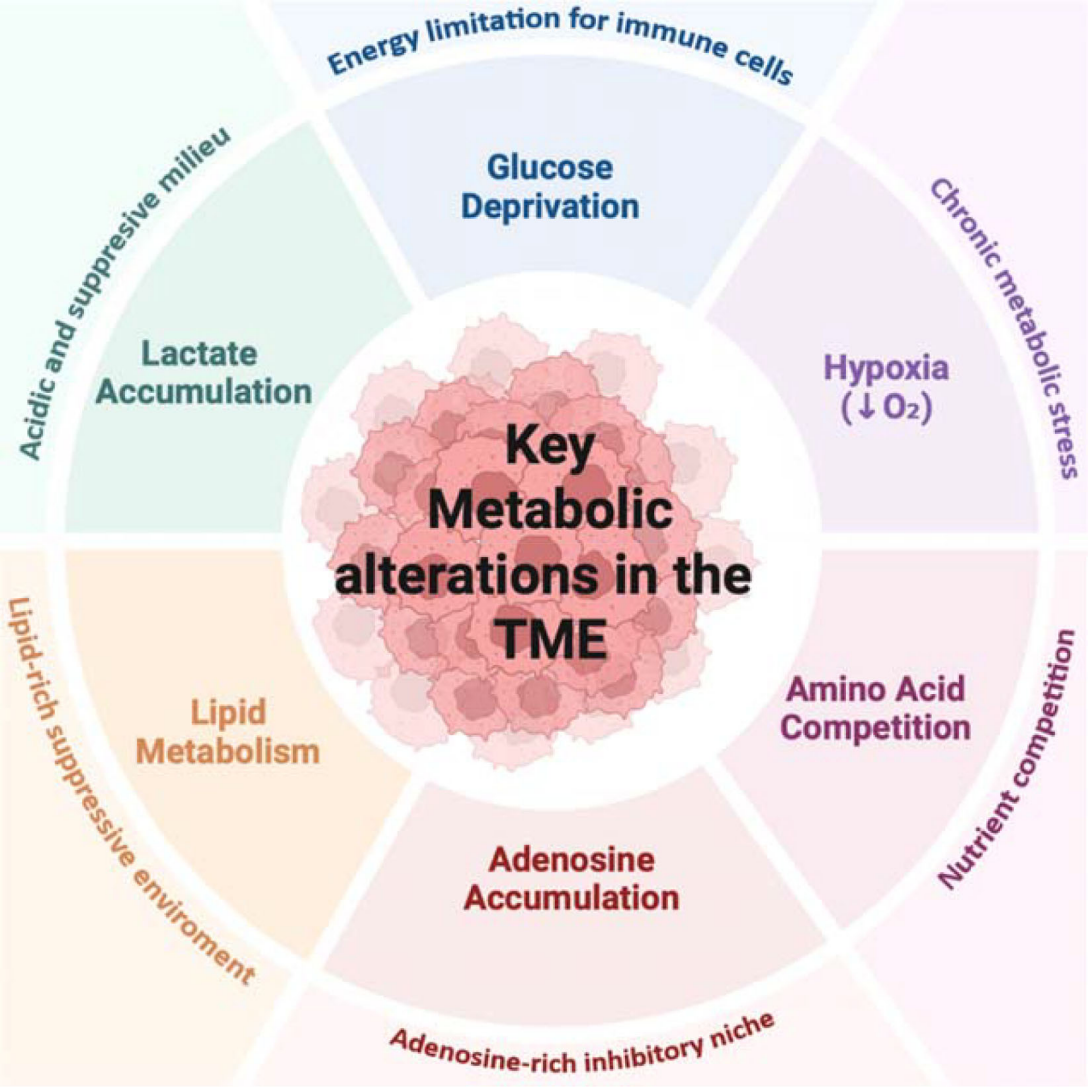
Key metabolic alterations in the TME. Schematic overview of major metabolic features of solid tumors, including glucose deprivation, hypoxia, amino acid competition, lactate accumulation, adenosine accumulation, and altered lipid metabolism. TME, tumor microenvironment.

Solid tumors are characterized by a harsh TME characterized by nourishment depletion, hypoxia, an acidic pH, waste product accumulation, and an environment that anti-cancer immune cells must overcome for tumor killing (**Figure 1**). Effective anti-tumor immunity relies on coordinated interactions between innate immune cells, particularly macrophages and NK cells, where macrophage-derived signals such as cytokines and metabolic cues are essential for optimal NK cell activation, cytotoxicity, and tissue recruitment. NK cells contribute to tumor control through direct cytotoxicity and cytokine production, while macrophages shape NK cell function by providing activating or suppressive signals depending on their polarization state. The immunosuppressive milieu that solid tumors produce, which restricts the cytotoxic potential of immune effector cells, is one of the primary obstacles to immunotherapy for solid tumors (8). The TME is additionally regulated by various cell types present at the tumor site, such as fibroblasts, endothelial cells, stromal cells, and immune cells, which, together with the extracellular matrix, make up the TME. The immune response is significantly weakened or even blocked by these environmental elements taken collectively, which offers a chance to increase the effectiveness of immunotherapies (9). Solid tumors have a variety of phenotypes with numerous subtypes among them, each with unique histological characteristics, development pathways, treatment responses, imaging characteristics, prognostic data, and patient outcomes. However, choosing an approach to treatment based on these traits is still difficult. Obtaining an ideal single classification that covers all conditions is probably hampered by TME heterogeneity and the possible inclusion of normal tissue in transcriptome studies (10, 11).

Solid tumors display significant variation in their metabolic profiles, which variably influences immune cell activities and intercellular interactions within the TME (12). Several characteristics of the TME are especially crucial for macrophage–NK cell crosstalk and a subsequent weakened or blocked NK cell response. Pancreatic ductal adenocarcinoma (PDAC) is defined by significant desmoplasia, inadequate vascularization, and extreme hypoxia, resulting in nutrient-deficient environments that severely hinder NK cell infiltration and cytotoxicity while promoting the accumulation of metabolically adapted, immunosuppressive macrophages. In these circumstances, the interaction between macrophages and NK cells is biased toward suppression since macrophages experiencing hypoxia and lipid stress reduce the expression of activating ligands and release inhibitory cytokines that constrain NK cell effector capabilities (13, 14). Conversely, glioblastoma arises in the lipid-dense milieu of the central nervous system, where increased lipid absorption and oxidation facilitate both tumor cells and tumor-associated macrophages (TAMs). Lipid-driven niches enhance macrophage morphologies that influence NK cell responses via modified cytokine production and receptor–ligand interactions, thereby transforming local immune surveillance (15, 16). In addition, melanoma is often linked to significant extracellular lactate accumulation due to increased glycolytic flux. Lactate-rich environments inhibit NK cell metabolism and cytotoxicity while concurrently fostering macrophage polarization toward immunoregulatory states, hence further impairing effective NK–macrophage communication (17, 18). The tumor type-specific metabolic programs collectively create unique spatial and biochemical niches within the TME, highlighting that variations in metabolic conditions differentially influence NK cell–macrophage interactions and should be considered when analyzing immune interactions in solid tumors.

Many immune cells can infiltrate tumors, and the clinical prognosis of cancer patients is closely linked to the composition and arrangement of these cells inside the TME. Current attempts to incorporate immunological markers into the traditional cancer prognostic classification systems have yielded promising outcomes. Our growing understanding of the TME has also led to the development of effective treatments for advanced cancer in recent years. TME analysis has proven essential in this context for predicting therapy response (19, 20).

## Tumor microenvironment

In the TME, while myeloid-derived suppressor cells (MDSCs) and Tregs inhibit tumor immunity, lymphocytes (including NK cells, dendritic cells, CD8+, and CD4+ helper T cells) and the proinflammatory macrophage subtype M1 induce an anti-tumor response at the immune cell level (21). Better outcomes for individuals with advanced solid tumors may be made possible by favorable TME reprogramming that limits immunosuppressive pathways and supports cellular immune function (21). At this point, it is imperative to understand the crosstalk between the immune cells in the TME. Numerous important characteristics of malignant tumors, such as angiogenesis, invasiveness, metastasis, control of the TME, and resistance to treatment, are directly or indirectly influenced by macrophages. There are two sides to macrophages in the TME. Macrophages, a common constituent of tumor stromal cells, can assemble around blood vessels, trigger angiogenesis, and encourage tumor invasion. However, they may potentially restructure the TME and phagocytose cancer cells (22).

In addition, studies utilizing NK-deficient mice have provided evidence of NK immunoediting, which shows that exposure to NK cells alters cancer immunogenicity, allowing the tumor to survive and grow in an immunocompetent environment. It is valuable to elucidate the communication between different cell populations, which is dominant in the TME, and to develop a successful treatment (23).

## Macrophage

Macrophages, the most prevalent immune cells in the TME, have a dual function in immunomodulation. Macrophages in malignancy are a diverse mixture ranging from pro-tumorigenic (M2) to anti-tumorigenic (M1). By releasing proinflammatory cytokines and displaying more immunostimulatory markers, classically activated macrophages (M1) support anti-cancer immunity. However, similar to the M2 phenotype, the majority of TAMs have been observed to exhibit poor antigen-presenting ability and elevated amounts of proangiogenic and anti-inflammatory cytokines (24, 25). Reducing the quantity of TAMs or reprogramming TAMs in the TME to the “effector” state is essential for cancer treatment because TAMs increase tumor development and metastasis. The immunoediting theory, which holds that the TME is a dynamically changing system, is supported by this evidence (23).

In addition to their phenotypic diversity, TAM-mediated immunosuppression is significantly enhanced by cytokine-driven signaling pathways, with the IL-10 axis being crucial. TAMs are a significant source of interleukin-10 (IL-10) inside the TME, and persistent IL-10 production activates the IL-10 receptor–signal transducer and activator of transcription-3 (STAT3) signaling pathway in both macrophages and adjacent immune cells, fostering an anti-inflammatory and tolerogenic condition (26, 27). The activation of STAT3 not only hinders macrophage maturation and antigen-presenting ability but also decreases the release of proinflammatory cytokines, notably IL-1α and IL-1β, thus reducing inflammasome-mediated immune activation (28, 29). The IL-10–STAT3–IL-1 regulation axis creates a feedback loop that reinforces immunosuppressive TAM phenotypes and restricts effective anti-cancer immune responses. In the setting of tumors, sustained STAT3 activation in TAMs facilitates angiogenesis, promotes tumor cell survival, and enhances metastatic spread, while concurrently inhibiting the recruitment and activity of cytotoxic lymphocytes (30, 31). These strategies collectively emphasize the crucial function of TAMs as active regulators of tumor progression, rather than just bystanders, and underscore the significance of cytokine-driven macrophage signaling pathways as essential factors in tumor immune evasion and treatment resistance.

In addition to IL-10, many different cytokines in the TME significantly influence the immunosuppressive characteristics of TAMs. Cytokines, including transforming growth factor-β (TGF-β), colony-stimulating factor-1 (CSF-1), interleukin-6 (IL-6), and interleukin-4/-13 (IL-4/IL-13), facilitate alternate macrophage polarization by enhancing STAT3- and STAT6-dependent transcriptional pathways. These signals limit antigen presentation, increase the expression of inhibitory ligands, and promote the development of pro-tumorigenic mediators that facilitate angiogenesis, matrix remodeling, and immune exclusion. This cytokine environment collectively creates a permanent immunosuppressive macrophage phenotype that collaborates with the IL-10–STAT3 pathway to maintain tumor immune evasion (32).

## NK cells

Several elements, including integrins, selectins, and chemokine receptors, regulate NK cell trafficking and homing, with NK cell migration to solid tumors being particularly influenced by the C-X-C motif chemokine receptor 3 (CXCR3) (33). CXCR3 is reported to be expressed by more than 60% of NK cells that infiltrate human breast cancer. Furthermore, NK cell infiltration of solid tumors has been mechanistically linked to the CXCR3 ligands CXCL9, CXCL10, and CXCL11, which are released in response to type I interferon (IFN) and interferon-gamma (IFN-γ) signaling.

NK cells also interact with macrophages in this process, and TAMs serve as an essential bridge via cell-to-cell contact and soluble mediator crosstalk. The TME significantly impacts the interactions between macrophages and NK cells, influencing their roles and overall anti-tumor responses. Investigating the vast communication networks between these two cell types can provide vital information to develop novel immunotherapeutic strategies (34, 35).

## Macrophage–NK cell crosstalk in the TME

Environmental obstacles working against optimal NK cell and M1 macrophage activity in solid tumors are due to hypoxia, immunosuppressive mediators, and metabolic factors. Solid tumors have extremely abnormal vasculature, with capillaries that are frequently leaky and have significantly reduced perfusion. An environment that is difficult for cell therapy to operate in is created by the inconsistent stroma and vasculature, which usually encourages hypoxic and acidic conditions (36). Due to intense intratumoral pressure, the typical solid tumor TME also restricts the penetration of cell therapy products, rendering a large portion of the tumor mass inaccessible and resulting in its untreated status (37). Immunosuppressive cytokines and suppressor cells, which may differ depending on the anatomic site and disease histology, are another reason the solid tumor TME poses immunologic difficulties. These TME conditions significantly influence the interactions between NK cells and macrophages, shaping their functions and overall anti-tumor responses (38).

## Metabolic factors

**Figure 2:**
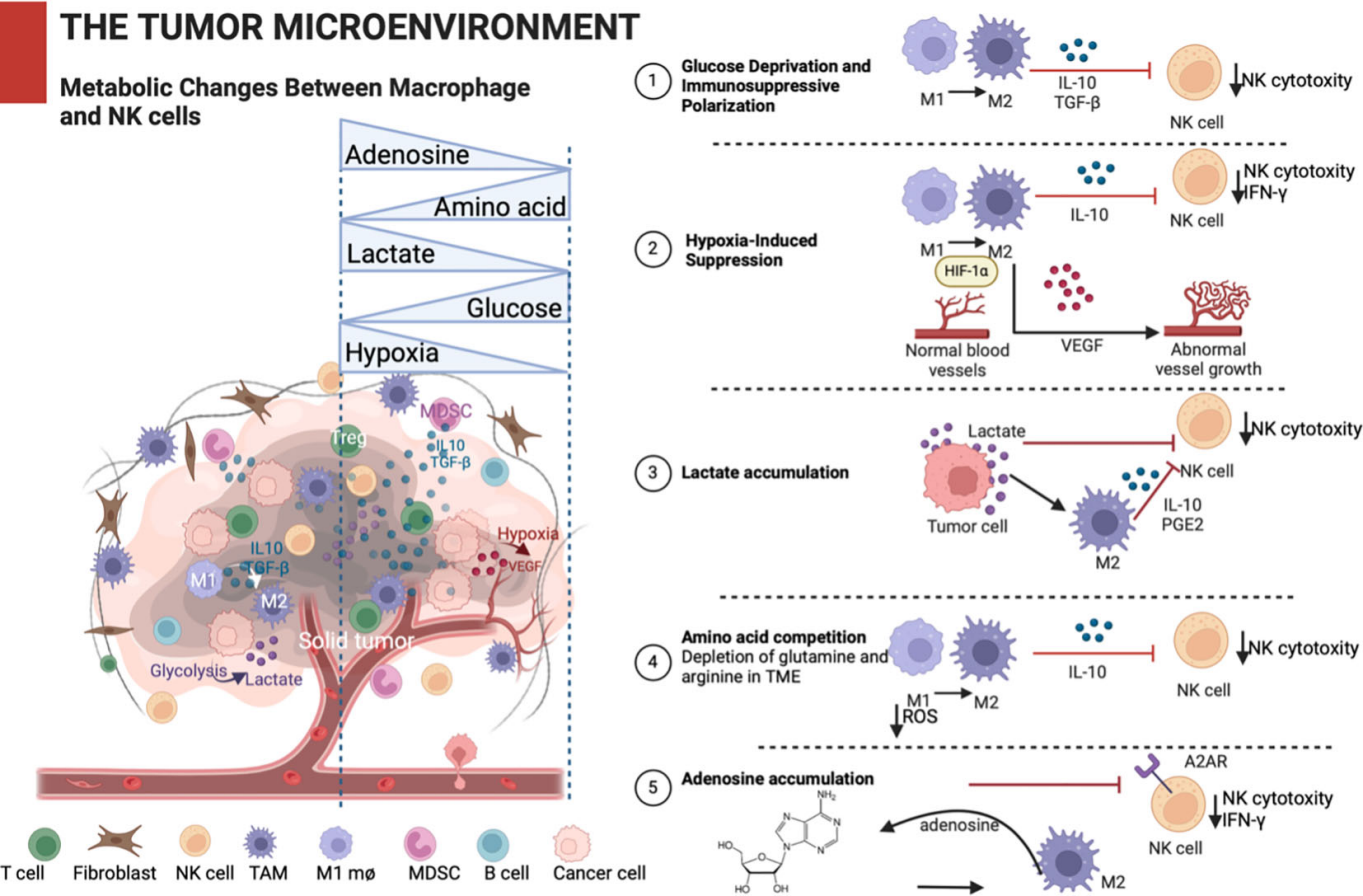
TME metabolic changes between macrophage and NK cells. Schematic representation of metabolic alterations in the solid TME and their impact on macrophage polarization and NK cell function. The tumor niche is characterized by hypoxia, glucose and amino acid deprivation, and accumulation of lactate and adenosine, which together promote immunosuppressive M2-like macrophage polarization and inhibit NK cell cytotoxicity. The right panels illustrate key metabolic mechanisms disrupting macrophage–NK cell cooperation. These metabolic stressors impair NK cell effector functions, reduce IFN-γ production, and reinforce immunosuppressive feedback loops that favor tumor progression (created by BioRender). TME, tumor microenvironment; NK, natural killer.

Once a tumor is established, cancer cells must overcome two major obstacles: first, they must secure sufficient nutrients to sustain rapid growth; second, they must evade host immune surveillance and attack. The distinct metabolic program of tumor cells can be utilized to address these issues. The metabolic environment within solid tumors impacts NK cells and macrophages individually and drives complex crosstalk between these two cell types, ultimately shaping the effectiveness of the anti-tumor immune response; key metabolic stressors and their differential effects on NK cells versus macrophages are summarized in **Table 1**; **Figure 2** (39).

**Table 1:** Metabolic stressors and their effects on NK cells *vs*. macrophages.

Metabolic stressor	Effect on NK cells	Effect on macrophages	Key pathways	Functional outcome on crosstalk
Glucose deprivation	↓ mTOR, ↓ IFN-γ, exhaustion	M2 skewing	mTORC1/mTORC2	NK suppression, TAM dominance
Hypoxia (↓O_2_)	HIF-1α → inhibitory receptors	HIF-1α → M2-like skewing	HIF axis	Immunosuppressive loop
Lactate accumulation	↓ Cytotoxicity, cytokine production	M2 polarization	GPR81	NK–TAM decoupling
Amino acid competition	↓ Cytotoxicity, ↓ IFN-γ production	Impaired NO production, IL-10 secretion, M2 skewing	mTOR, ARG1, iNOS	NK suppression, immunosuppressive loop
Adenosine	A2A-mediated inhibition; ↓ IFN-γ, ↓ cytotoxic granules; impaired OXPHOS and glycolysis	M2 polarization	cAMP–PKA	NK paralysis
Accumulation				
Lipids	Metabolic overload; impaired cytotoxic function	FAO-driven M2 polarization; immunosuppression	CD36–FAO; SREBP1	Pro-tumor metabolic loop, NK suppression

NK, natural killer; mTOR, mechanistic target of rapamycin; TAM, tumor-associated macrophage; HIF-1α, hypoxia-inducible factor-1α; NO, nitric oxide; ARG1, arginase-1; iNOS, inducible nitric oxide synthase; FAO, fatty acid oxidation.

## Metabolic competition as a central regulator of NK cell–macrophage function in the TME

The TME is marked by significant metabolic competition for vital nutrients, including glucose; amino acids like arginine, tryptophan, and glutamine; fatty acids; and oxygen (40). This competition significantly influences the behavior and functional capacity of innate immune cells, especially NK cells and macrophages, by restricting availability to metabolites essential for effector functions. Rapidly proliferating tumor cells demonstrate elevated glucose uptake and aerobic glycolysis, often exceeding NK cells in glucose availability. Consequently, glucose deprivation in NK cells inhibits mechanistic target of rapamycin (mTOR) signaling and glycolytic reprogramming, resulting in decreased cytotoxicity, altered IFN-γ production, and lower anti-tumor efficacy (41). Moreover, macrophages promote immune suppression through amino acid competition. TAMs often have increased levels of arginase-1 (ARG1), resulting in localized arginine depletion inside the TME (42, 43). Arginine is essential for NK cell proliferation and nitric oxide (NO) production by macrophages; hence, ARG1-mediated arginine depletion reduces NK effector functions and hinders a critical cytotoxic mechanism of M1 macrophages (42, 43). Alongside arginine metabolism, altered tryptophan catabolism via indoleamine 2,3-dioxygenase (IDO) and competition for glutamine provide metabolically restricted conditions that foster immunosuppressive myeloid phenotypes and reduce NK cell responsiveness.

Taken together, these pathways driven by nutrient competition demonstrate the metabolic interconnection of tumor cells, NK cells, and macrophages inside the TME. Immune suppression results from a coordinated breakdown of metabolic communication, wherein tumor cells monopolize essential resources, leading to the progressive reprogramming of macrophages and NK cells into non-cytotoxic, immunosuppressive states. Metabolic competition is a fundamental factor in immune dysfunction in solid tumors and serves as a crucial conceptual foundation for comprehending innate immune suppression inside the TME (44).

## Glucose deprivation and immunosuppressive polarization

In mammalian cells, glucose is a primary energy source that produces adenosine triphosphate (ATP) via oxidative phosphorylation and glycolysis. Additionally, it is a precursor to lipids, nucleotides, and amino acids. A characteristic of cancer cell development is the increased reliance on glucose to drive aerobic glycolysis (the Warburg effect), which increases the production of cellular metabolites needed for biomass formation and to support nutritional signaling. Warburg explained a process wherein cancer cells preferentially utilize glycolysis to produce ATP, even when oxygen is present. Tumor-driven glucose restriction in the TME may decrease NK cell glycolysis, hindering their anti-tumor activity (45, 46). A study by Cong et al. examined NK cells in a lung cancer mouse model. They discovered that NK cells from the lung cancer microenvironment had reduced glycolytic rates and decreased cytotoxicity and cytokine production (47). The highly conserved mTOR kinase, which is present in one or both of the protein complexes mTORC1/mTORC2, is one of the primary glucose-sensing pathways. A key metabolic regulator that supports the glycolytic pathway, among other processes, may also be beneficial. As the catalytic subunit of the mTORC1 and mTORC2 complexes, mTORC1 is primarily regulated by hypoxia, growth hormones, nutrition, and energy, among other factors, and is crucial for cell survival, autophagy, and proliferation. When glucose levels are normal, mTORC1 is triggered, encouraging cell division and growth. Autophagy is triggered to increase cell survival, and mTORC1 activity is inhibited in low-glucose environments (48).

It has been suggested that mTORC1 is a therapeutic target for several tumors since it is consistently hyperactive in these conditions and because of its pro-anabolic characteristics. Even though several mTORC1 inhibitors have been tested on a variety of cancer types, their use in clinics is now limited due to poor performance. This is also probably explained by the finding that mTORC1 inhibition protects tumor cells against glucose deprivation, a typical occurrence in the TME (49).

mTOR, Rictor, GβL, PRR5, Deptor, and SIN1 make up mTORC2, whereas mTOR, Raptor, GβL, and Deptor make up mTORC1. To stimulate cell growth when energy is abundant and catabolism when the body is starving, mTORC1 combines signals from several growth factors, nutrients, and the energy supply. Whereas mTORC2 primarily governs cell survival and proliferation, mTORC1 regulates cell growth and metabolism. Phosphoinositide-3-kinase (PI3K)/AKT, tuberous sclerosis complex subunit 1 (TSC1)/tuberous sclerosis complex subunit 2 (TSC2)/Rheb, LKBL/adenosine 5′-monophosphate-activated protein kinase (AMPK), VAM6/Rag GTPases, and numerous other signaling pathways have been linked to mTOR, according to studies (50). By controlling immune cell development and function, the mTOR pathway—frequently triggered in tumors—promotes tumor progression. The investigation of novel immunotherapies and the advancement of tumor treatment will benefit from this. According to Zhihua et al., gastric cancer (GC) dramatically decreased the expression of microRNA (miRNA)-30c. Subsequent research revealed that the expression of miRNA-30c was decreased by hypoxia-inducible factor-1α (HIF-1α). In TAMs, downregulating miRNA-30c will decrease glycolysis and mTOR activity. Preventing M1-type macrophage formation and function will ultimately encourage GC growth and metastasis (50, 51).

Caumanns et al. tested the mTORC1/2 inhibitor AZD8055 in ovarian clear cell cancer (OCCC) cell lines after traditional platinum treatment failed to treat OCCC. The OCCC cell line was found to be sensitive to AZD8055, and AZD8055 was confirmed to work in a xenotransplantation model (52). Similarly, Hanna et al. showed that everolimus worked well for patients with undifferentiated thyroid carcinoma who had PI3K/mTOR/Akt mutations (53).

Many researchers have highlighted the importance of mTOR for metabolic reprogramming, NK cell activation, and the detrimental effects of mTOR suppression on NK cell function. NK cell metabolism and function are inhibited by TGF-β, which can restrict mTOR, which is sensitive to nutrient availability. Therefore, it is likely that mTOR may be suppressed in the nutrient-deprived TME, where there is also a greater production of TGF-β, thereby reducing the action of NK cell effectors. This inhibition may further restrict NK cell metabolism, limiting their ability to mount anti-tumor responses. The resulting decreased NK cell activity (due to less NK cell-mediated killing) indirectly promotes the accumulation of immunosuppressive M2-like macrophages (50).

Additionally, low glucose favors the M2 polarization of macrophages. M2-like TAMs then secrete immunosuppressive cytokines, such as TGF-β and IL-10, which directly suppress NK cell activities, reducing their cytotoxicity further and impairing their ability to recognize tumor cells. Recent studies show that the metabolic polarization and functional polarization of TAMs are tightly linked to changes in glucose metabolism. M2-polarized macrophages rely more on mitochondrial metabolism through the tricarboxylic acid (TCA) cycle and increase fatty acid oxidation (FAO) to suppress anti-tumor immunity and promote tumor metastasis, whereas M1 macrophages switch from oxidative phosphorylation to increased glycolytic and pentose phosphate pathway (PPP) activities for effective activation and inflammation (54). Significant glucose uptake and consumption by TAMs result in the acquisition of pro-tumor M2-like properties. This creates a feedback loop in which glucose deprivation limits NK cell function and supports macrophage-driven immunosuppression. Characterizing the active remodeling of glucose metabolism in tumor-promoting TAMs and the role of immune-metabolic regulatory networks in TAMs in determining tumor development and treatment responsiveness is crucial.

The metabolic reprogramming of M1 macrophages is directed toward the flux of the pentose phosphate pathway concerning the augmentation of glycolysis and fatty acid synthesis, which includes the Krebs cycle, the production of reactive oxygen species (ROS), and citrate efflux. Through the activation of NADPH, succinate, and PGE2 synthesis, ROS and mitochondrial production stabilize HIF-1α (55, 56).

In mouse lung and colorectal cancer models, M2 transmission to the M1 phenotype was preferred by FAO suppression. In certain mouse TAMs, MIF, released by cancer cells, promotes lung carcinogenesis by upregulating fatty acid synthase (FASN). Furthermore, TAMs invading breast cancer have elevated expression of FABP5, which is linked to the accumulation of lipid droplets and the release of immunostimulatory cytokines, such as IL-6. In late-stage breast cancer, FABP4 is elevated in macrophage infiltration, which promotes tumor growth by promoting IL-6 production via signaling from the NFκB–miR-29b pathway (57, 58).

In addition, chemotherapeutic stress, oxygen tension, malnutrition, and metabolite buildup encourage the release of CSF1, VEGF, and IL34 to reeducate TAMs, favor vascular dysfunction and neoangiogenesis, and reduce antigen presentation. By encouraging phosphoglycerate kinase 1 (PGK) phosphorylation, TAMs release IL-6 to promote glycolysis, promoting aerobic glycolysis and carcinogenesis (56).

## Hypoxia-induced suppression of NK cell activity

Most solid tumors have disordered vascularization and high oxygen consumption, which results in the formation of hypoxic areas either permanently or temporarily. The hypoxia-inducible family of transcription factors (HIFs), which affect many genes, helps cells adapt to these hypoxic environments. Most NK cells’ dysregulated genes under hypoxia are specifically linked to metabolic and biosynthetic processes, which aligns with findings in other cells where HIF-1α supports or encourages glycolytic metabolism. Short-term hypoxia and IL-15 priming work together to increase the expression of genes related to the glycolytic pathway in human NK cells (59). Hypoxia tends to increase the expression of immunosuppressive molecules, pushing these immune cells into a less effective, often dysfunctional state. The survival and activity of NK cells and macrophages can be impacted by the frequently hypoxic nature of solid tumors. Immunosuppressive factors can be expressed more when hypoxia is present, which can cause these immune cells to fail. Numerous immunosuppressive cells that disrupt NK cell activities are seen in hypoxic tumor locations. These include TREG cells, M2-like TAMs, and MDSCs (60). Each cell type creates an environment that limits NK cells’ ability to attack tumor cells, often enhancing the tumor’s ability to evade immune responses. Notably, NK cells have downregulation of activating receptors such as NKp30, NKp46, or NKG2D, and the alteration of genes linked to immunomodulatory functions also contributes to the reduced effector functions under hypoxic conditions. Additionally, hypoxia facilitates tumor immune evasion via additional pathways, such as the degradation of granzyme B, a key molecule in NK cell-mediated tumor cell lysis (45).

This complex hypoxic environment thus weakens NK cell functionality while allowing tumors to evade immune surveillance effectively. However, hypoxia in the TME enhances macrophage differentiation toward an M2-like, pro-tumor phenotype through HIF-1α signaling. These M2 macrophages release high levels of IL-10, inhibiting NK cell activation and decreasing IFN-γ production by NK cells, further impairing NK cell-mediated anti-tumor activity (54). The weakened NK cell activity in a hypoxic environment increases macrophage infiltration and M2 polarization, as NK cells can less suppress these cells through cytotoxic killing. This hypoxia-induced shift in macrophage phenotype also promotes the release of vascular endothelial growth factor (VEGF) by TAMs, which worsens hypoxia by driving abnormal blood vessel formation. Thus, hypoxia establishes a cycle where macrophages promote tumor-supportive angiogenesis and inhibit NK cell function through cytokine signaling and physical barriers (61–63).

To drive conditional lentiviral gene expression only under hypoxic circumstances, a new study modified human macrophages to produce genes regulated by hypoxia-sensitive elements in the lentiviral promoter region. They examined the temporarily increased expression of reporter genes and the released cytokine interleukin-12 (IL-12) in transduced macrophages cultured under hypoxic environments. Under hypoxic conditions *in vitro*, it was demonstrated that modified macrophages conditionally and transiently express lentivirally encoded gene protein products, such as IL-12. When normoxic circumstances were restored, the expression of lentiviral payloads recovered to its baseline levels. In both the hypoxic xenograft model of glioblastoma and the presence of human colorectal cancer cells, reporter genes regulated by hypoxia response elements were increased under hypoxic circumstances. Cell engineering studies that will turn the tumor system into an advantage in tumor immunotherapy studies are very valuable (64).

## Lactate accumulation and M2 polarization

Tumor cell metabolism produces excessive lactate, inhibiting NK cell cytotoxicity by lowering granule content and cytokine production. Lactic acid inhibits the nuclear factor of activated T cells (NFAT) activity, which is essential for IFN-γ transcription. Therefore, their ability to produce IFN-γ is compromised when NK cells are exposed to it. The primary cause of lactate accumulation is tumor metabolic reprogramming, which occurs when cancer cells prefer glycolysis over oxidative phosphorylation. Lactate also encourages macrophage M2 polarization, which produces anti-inflammatory signals that suppress NK cell activities. Hypoxia and oncogenes trigger this alteration. Lactate-stimulated M2-polarized macrophages afterward release more immunosuppressive substances, including prostaglandin E2 (PGE2) and IL-10, producing a lactate-driven loop that sustains a macrophage phenotype that promotes tumor development while persistently inhibiting NK cell function (65, 66). One indicator of tumor metastasis and overall survival is the generation of lactic acid (67). The intracellular and extracellular lactic acid concentrations determine how much lactic acid is secreted by monocarboxylate transporter 4 (MCT4) in cancer cells. The effects of extracellular lactate include limiting monocyte conversion to dendric cells (DCs), decreasing monocyte motility and cytotoxic T lymphocyte (CTL) activity, and lowering cytokine release from DCs and CTLs. Furthermore, tumor cells’ release of lactic acid into the extracellular space can prevent immune cells from secreting lactic acid, which results in cell death from too much intracellular lactic acid (68, 69).

## Amino acid competition and cytokine modulation

The depletion of key amino acids like glutamine and arginine in the TME affects both NK cells and macrophages. NK cells have diminished cytotoxic capabilities due to an absence of amino acids, whereas arginine deficiency within the TME directly undermines an essential anti-tumor activity of M1 macrophages, specifically NO synthesis. The generation of NO by inducible nitric oxide synthase (iNOS) is entirely dependent on arginine; thus, diminished arginine availability inhibits M1 macrophages from performing their cytotoxic and tumoricidal roles. The reduction of NO-mediated effector activity exemplifies a distinct and mechanistically characterized instance of metabolic crosstalk failure within the TME. Additionally, macrophages reacting to amino acid deficiency may elevate the release of immunosuppressive cytokines, such as IL-10, further impeding NK cell activities. Amino acid competition simultaneously diminishes NK cell cytotoxicity and impairs macrophage-mediated tumor destruction, shifting both cell types toward an immunosuppressive state and compromising efficient anti-tumor immunity (54, 70).

## Adenosine accumulation and suppressive feedback

Hypoxia encourages ATP and adenosine monophosphate (AMP) release; together with those, it releases ectonucleotidases to convert ATP to ADP and ADP to adenosine. High adenosine levels in the TME suppress NK cell cytotoxicity by engaging A2A receptors, which reduce IFN-γ and cytotoxic granule production (59). This NK cell suppression allows M2 macrophages to proceed unchallenged. Adenosine also stimulates macrophage M2 polarization, boosting adenosine synthesis and producing an adenosine-rich, immunosuppressive environment. The ongoing adenosine synthesis produces additional M2 macrophages, further inhibiting NK cell activities and strengthening an immunosuppressive feedback loop. Adenosine suppresses the function of NK cells activated by IL-12/15 by blocking mitochondrial oxidative phosphorylation (OXPHOS) and glycolytic activity (71). Adenosine blockage represents a promising cancer immunotherapy approach that targets the immunosuppressive adenosine pathway within the TME to activate the body’s immune response against cancer. These strategies aim to restore T-cell function, promote NK cell activities, and improve the efficiency of other treatments, such as checkpoint inhibitors, by inhibiting critical enzymes (CD39 and CD73) that create adenosine or by blocking adenosine’s receptors (A2A and A2B) (72, 73).

ROS are prevalent in solid tumor TMEs and facilitate disease development through several pathways, including immunoevasion. Significantly, ROS impose damage on non-transformed cells and immune effector populations, including NK cells, which exhibit heightened sensitivity to ROS-induced genotoxic stress and apoptosis. In contrast, malignant cells and immunosuppressive M2-like TAMs often enhance antioxidant and detoxifying pathways, allowing them to endure oxidative stress. This selective vulnerability, wherein ROS preferentially harm anti-tumor effector cells while preserving tumor cells and suppressive myeloid populations, constitutes a pivotal and empirically substantiated mechanism of metabolic immune evasion within the TME and should be regarded as a hallmark of redox-mediated immune suppression in solid tumors (71).

In solid tumors, NK cells are found close to monocytes and macrophages that have infiltrated the tumor. Tumor-infiltrating monocytes’ and macrophages’ physiological responses in the TME are closely regulated by the communication between NK cells and these cells, which can either activate or repress the immune system (74). Tumor-associated NK cells (TA-NKs) and TAMs have a complex and dynamic relationship. Thus, direct cell-to-cell contact and the release of specific factors by activated macrophages may help reactivate NK cells’ anti-tumor functions in the TME. Also, understanding the mechanisms behind this metabolism regulation in the TME may lead to new possibilities for developing highly effective immunotherapies and drug discoveries.

## Lipid metabolism

For tumor cells to adjust to the TME, aberrant lipid metabolism is not negligible. It has a major impact on the quantity and functionality of immune cells, such as T cells, dendritic cells, TAMs, and suppressor cells produced from bone marrow. It is commonly recognized that the TME suppresses the immunological response, and lipid metabolism plays a key role in this process (54). The metabolism of glucose alone is insufficient to power tumor cells. Therefore, tumor cells use several methods to enhance lipid metabolism to meet the need for rapid growth and proliferation. First, tumor cells can compete with other cells for lipids from the TME due to increased expression of lipid uptake-related proteins such as low-density lipoprotein receptors (LDLRs), CD36 (fatty acid translocase), and fatty acid-binding proteins (FABPs). Second, it has been discovered that lipid oxidation enzyme-related transcription factors are highly expressed. Third, tumor cells are capable of producing lipids on their own. Lipids give tumor cells energy but also function as signal transmission molecules and the building blocks of their cellular structure (75, 76). The amount of lipid metabolism activity varies between pro-tumor and anti-tumor immune cells; pro-tumor immune cells, like M2-macrophages and Treg cells, typically have more active lipid metabolism, whereas anti-tumor immune cells prefer to obtain energy through glucose metabolism (70).

Lipid metabolic reprogramming, particularly the dynamic balance between fatty acid synthesis (FAS) and FAO, is a characteristic of many malignant tumors and is essential for developing malignancies. Studies have shown that the metabolism of long-chain fatty acids, mainly unsaturated fatty acids (UFA), is responsible for the immunosuppressive phenotype of TAMs (75, 77). TAMs mechanically overexpress the scavenger receptor CD36 to obtain sufficient energy and guarantee cellular survival. This speeds up the transportation of FAs to accumulate lipids and use FAO; increased FAO causes a high rate of oxidative phosphorylation and the STAT6 signaling pathway, which controls gene transcription to determine the function of TAMs (78–80). In malignancies, overexpression of lipogenic enzymes, including FASN and acetyl-CoA carboxylase (ACC), is frequently observed and linked to an undesirable prognosis (81). The identification of enriched genes related to lipid metabolism, including targets of the transcription factor sterol regulatory element-binding protein 1 (SREBP1), suggests that significant metabolic reprogramming, a necessary condition for macrophage alternative activation, may be connected to the shift from early to late tumor development. SREBP1 significantly contributes to the resolution phase of TLR4-induced gene activation by modifying macrophage lipid metabolism. By inducing SREBP1, mTOR signaling promotes FAS, triggering FASN and ACC (70).

The lipid-rich milieu created by FAO-dependent TAMs significantly affects adjacent NK cells. In contrast to macrophages, NK cells are physiologically tailored for glycolysis to maintain cytotoxicity and cytokine synthesis, while an abundance of lipids in the TME has been demonstrated to interfere with this metabolic pathway. Enhanced fatty acid absorption by NK cells, partially facilitated by CD36, results in intracellular lipid buildup, mitochondrial strain, and compromised mTOR signaling, culminating in diminished IFN-γ synthesis and cytotoxic activity (82, 83). Moreover, oxidized lipids and lipid peroxidation byproducts produced in lipid-dense tumor environments intensify NK cell dysfunction by inducing oxidative stress and metabolic exhaustion. The TAM-mediated lipid remodeling of the TME preferentially promotes fatty acid oxidation-dependent immunosuppressive macrophage phenotypes while metabolically limiting glycolysis-dependent NK cells, thus enhancing immune suppression in solid tumors.

## Metabolically regulated soluble factors between NK and macrophages

The development and activity of NK cells may be closely regulated by the proinflammatory and immunosuppressive cytokines produced by macrophages, a major mediator in the TME. For instance, macrophage-secreted IL-12/-15/-18 can “train” NK cells in the “prime” condition. Thus, NK cell-derived cytokines such as IFN-γ, TNFα, and GM-CSF shape macrophage responses within the TME, affecting IL-12 and IL-18 secretion with both immunostimulatory and immunoregulatory outcomes, as summarized in **Table 2** (84).

## IL-12 and IFN-γ loop

A practical, proinflammatory type 1 cytokine, IL-12, has long been studied as a possible cancer immunotherapy. One of the primary sources of IL-12 production is macrophages (85). By encouraging IFN-γ synthesis, lytic activity, and NK cell proliferation in a range of tumor types, IL-12 strengthens anti-cancer immune responses. IFN-γ, which is quickly secreted by NK cells in response to IL-12, activates macrophages early in an immune response. Moreover, IL-12 is the primary cytokine promoting T helper 1 cell development and causes T cells to produce IFN-γ (86).

The proinflammatory, M1-like phenotype that this IFN-γ induces in macrophages subsequently improves their anti-tumor activities by increasing their capacity to phagocytize tumor cells. IL-12 also boosts the production of chemokines such as CXCL9, CXCL10, and CXCL11, encouraging macrophage polarization toward the M1 (proinflammatory) phenotype. These chemokines help attract NK and T cells to the tumor location by binding to their CXCR3 receptor. This cycle strengthens the immune system and stimulates both cells to take on more aggressive roles in the fight against tumor cells. Consequently, it has been shown that IL-12 works in tandem with other soluble factors released by macrophages, such as IL-2, IL-15, and IL-18. To sufficiently prime NK cells for IL-12 signaling responsiveness, both IL-15 and IL-18 increase the expression of IL-12R (IL-12Rβ1 and β2) on NK cells (84). It was previously demonstrated that IL-12 administered systemically resulted in dose-limiting effects.

Nevertheless, cancer researchers are still drawn to IL-12 because of its pleiotropic activity, which involves activating several effector systems and reversing tumor-induced immunosuppression. Growing interest is in developing methods that optimize IL-12 delivery to the TME while reducing systemic exposure. Various IL-12 delivery methods, including polymeric nanoparticles and immunocytokine fusions, have shown strong anti-tumor immunity with fewer side effects in preclinical investigations (87). Several localized IL-12 delivery strategies have recently advanced to the clinical stage, and several more are on their way to being adopted. When combined, targeted delivery methods have promoted an increase in IL-12 that could ultimately enable this potent cytokine to reach its significant therapeutic potential (85).

Further encouraging IL-18 signal transduction is the ability of IL-12 or IL-15 to upregulate the expression of IL-18Rβ. In addition, IL-12, IL-23, and IL-27, which belong to the IL-12 cytokine family, play dual roles in enhancing anti-tumor immunity and promoting immune suppression under certain conditions. The subunits IL-12p40 and IL-12p19 combine to form the heterodimer known as IL-23. On the surface of NK cells, IL-23 increases the production of IL-23R, which phosphorylates STAT3/4 and causes NK cells to produce IFN-γ. Additionally, M1-type macrophages release IL-23 and IL-1β, which both synergistically function together to upregulate activating receptor NKG2D on NK cells and enhance NK cells’ ability to kill cancer cells. Also, NK cell growth and function are directly related to the transcription factor T-bet, whose expression is regulated by IL-23 (87, 88).

Proinflammatory cytokines are linked to advanced cancer stages, immunotherapy resistance, and poor prognoses, including overall and progression-free survival, objective response, and disease control rates (89). Inflammatory indicators triggered by IFN-γ, such as circulating neopterin and the kynurenine-to-tryptophan ratio (KTR), are elevated in cancer patients and predict a poor prognosis (90). A higher level of STAT3 activity is linked to advancing head and neck squamous cell carcinoma (HNSCC) (91). The results show that STAT3 can be used as a target for immunotherapy, which has proven very beneficial for treating cancer, as well as a biomarker for prognosis and sensitivity prediction (92–95).

## IL-15

As a “priming factor”, interleukin-15 (IL-15) promotes the recruitment, activation, proliferation, survival, and cytolytic activity of macrophages and/or NK cells. Macrophages can secrete IL-15, essential for NK cell survival, proliferation, and cytotoxic activity. IL-15 increases the transmission of downstream PI3K-Akt-mTOR signals. Consequently, it controls the expression of genes linked to the mitochondrial respiration profile and NK cell metabolism.

It is commonly accepted that IL-15 uses the Janus kinase (JAK)–STAT pathway to carry out downstream signal transduction. By activating Janus kinase 2 (JAK2), IL-15 also stimulates NK cells. Increased IL-15 availability can boost NK cell numbers and enhance their ability to kill tumor cells, indirectly supporting macrophage anti-tumor activity. This signaling pathway supports NK cells’ energy demands and functional capacity, promoting their metabolic fitness and effectiveness within the TME (65, 96). Accordingly, IL-15 has emerged as a promising therapeutic agent to boost NK cell metabolic fitness and cytotoxic function in cancer immunotherapy settings (96, 97).

## IL-10 and TGF-β: suppressive cytokines

There are suppressive cytokines that modulate immune cells in the TME, like IL-10 and TGF-β. IL-10 is primarily secreted by tumor cells and TAMs. IL-10 increases tumor-resident tumor antigen-specific CD8+ T cells through STAT1 activation and IFN-γ production while suppressing a high level of inflammatory response by activating Janus kinase 1/signal transducer and activator of transcription-3. IL-10 inhibits macrophage function by downregulating membrane molecules (MHC II molecules and CD80/CD86 costimulatory molecules) and soluble factors (including nitric oxide, IL-12, and IFN-γ) (98, 99).

In patient samples from various cancer types, it has been shown that NK cell activities and IL-10 are negatively correlated. Low levels of IL-10 inhibit macrophages from producing TNFα, IL-12, IL-15, and IL-18, which affects NK cell activation and function (84). This creates a more immunosuppressive environment, leading NK cells to a less active, less cytotoxic state and supporting the pro-tumor functions of macrophages. Such cytokines can help tumors evade immune surveillance (100).

Recent therapeutic approaches are being developed to modulate these specific cytokines. In preclinical models, IL-10 treatment and IL-10 signaling blockage have shown promise in reducing tumor growth. These contradictory results indicate that IL-10’s function depends on the environment and the cell. In solid tumors, there is growing evidence that intratumoral IL-10 coordinates a pro-tumoral and immunosuppressive TME, offering a therapeutic justification for blocking. According to these pathways, immune cells that reside in or traffic into the TME may require functional reprogramming, which may be achieved by persistently blocking IL-10 (101–104).

In human colorectal cancer liver metastasis cultures, αIL-10 increased T cell-mediated cancer cell killing by 1.8 times. αIL-10 raised the fraction of CD8+ T cells without exhaustion transcription alterations and the expression of macrophages with the human leukocyte antigen-DR isotype (HLA-DR). The crucial function of antigen-presenting cells was confirmed when primary histocompatibility complex class I or II (MHC-I or MHC-II) inhibition reduced the anti-tumor effects of αIL-10. Myeloid cell-mediated immunosuppression of murine CAR-T cell growth and cytotoxicity was also prevented by blocking IL-10 signaling. Both as a standalone treatment and to improve the function of adoptively transferred CAR-T cells, neutralizing the effects of IL-10 in human colerectal cancer liver metastase (CRLM) offers therapeutic potential (104).

In addition to IL-10, TGF-β is another well-known immunosuppressive cytokine produced mainly by tumor cells and TAMs. TGF-β causes IL-10 to be upregulated and activates cytokines, including TNFα and IL-12, to be reduced; macrophages shift toward the M2-type phenotype (105). TGF-β promotes monocyte survival in myeloid cells by upregulating the production of the transcription factors RUNX1 and SOX4 and the tumor necrosis factor ligand TNFSF14, which collectively prevent apoptosis and promote cell survival (106). TGF-β is necessary for the development of alveolar macrophages and Langerhans cells and for the preservation of mature cell pools, where autocrine signaling is crucial. During their formative stage, some subsets of macrophages appear to be less dependent on the effects of TGF-β activity. However, TGF-β does affect their phagocytic activity, specifically by downregulating class A scavenger receptors and CD36. By acting as a chemo-attractant for monocytes, TGF-β promotes the production of integrins in these cells, which have an affinity for collagen type IV, laminin, and fibronectin and help the cells embed in the extracellular matrix of tumors. TGF-β also promotes the release of matrix metalloproteinases, which break down the surrounding tissues and make it easier for immune cells like monocytes to enter tumors. There, TGF-β promotes monocyte development into macrophages, and its function on this myeloid subgroup changes from proinflammatory to immunosuppressive (107).

TGF-β also inhibits NK cells’ ability to fight tumors. Reduced expression of the natural cytotoxicity receptor NKp30, the NK glycoprotein DNAM-1, and the Killer Cell Lectin Like Receptor (NKG2D) in the presence of TGF-β reduces the ability of NK cells to engage cancer cells (108). By suppressing the expression of NK activating receptors (such as NKG2D and NKp30), secreting proinflammatory cytokines (such as IFN-γ), or increasing the expression of inhibitory receptors (such as ILT-2, also known as CD85j), TGF-β prevents the growth, maturity, or function of tumor-infiltrating NK cells (109). TGF-β causes IL-10 to be upregulated and activating cytokines (including TNFα and IL-12) to be reduced, and macrophages shift toward the M2-type phenotype (110).

Numerous anti-cancer pharmacological treatments target particular TGF-β signaling pathway mediators or TGF-β activators, which have shown auspicious outcomes in preclinical animal models or have been investigated in human clinical trials (111–113).

Many strategies have been implemented to disrupt the TGF-β signaling activation in cancer. To put it briefly, the therapeutic medicines that are currently available can target the kinase domain of TGF-β receptors, ligand–receptor interactions, the bioavailability of TGF-β, or other signaling molecules (114). They can be found as antisense oligonucleotides, ligand traps, neutralizing antibodies, or small-molecule inhibitors (115). However, none of these therapy modalities have completed phase II clinical trials because of serious side effects or a lack of therapeutic effectiveness (116). TGF-β target agents can cause a variety of toxicities, including skin lesions, cutaneous squamous cell carcinomas, basal cell carcinomas, eruptive keratoacanthomas, and hyperkeratosis, as well as cardiac toxicity, which includes inflammatory, degenerative, and hemorrhagic lesions in the heart valves (107, 117). Although small-molecule kinase inhibitors are linked to cardiovascular toxicity, ligand traps and antibodies are more likely to cause skin toxicity (118). The toxicities seen mainly depend on the therapeutic agent used to target TGF-β (111). Notably, given that TGF-β functions as a tumor suppressor, it will be crucial to determine whether therapies targeting TGF-β will raise the risk of secondary cancers and skin neoplasias (119). Consequently, we must better understand the molecular mechanisms via which TGF-β signaling regulates both healthy and cancerous activities (120). To minimize the (cardiac) adverse effects, galunisertib (LY2157299) has been used as a medication treatment regimen with a schedule of 2 weeks on and 2 weeks off. Predictive biomarkers may also help identify patients who will benefit the most from anti-TGF-β medication. A poor prognosis is associated with high expression of TGF-β target genes in cancer patients with mesenchymal subtypes, according to transcriptional profiling of samples from patients with a wide range of cancer types, including glioblastoma, pancreatic cancer, breast cancer, ovarian cancer, colorectal cancer, and non-small cell lung cancer. Therefore, anti-TGF-β therapy may be especially beneficial for subgroups of patients with malignancies that have a mesenchymal phenotype (111, 121).

**Table 2:** Soluble mediators and their effect in TME.

Mediator	Source	Target	Functional effect
IL-12	M1 macrophage	NK cells	NK recruitment and activation
IL-10	M2 macrophage	NK cells	NK inhibition
IL-15	Macrophage (trans-presentation)	NK cells	NK survival, proliferation, and cytotoxicity
TGF-β	TAMs	NK cells	NK exhaustion
IFN-γ	NK cells	TAMs	TAM reprogramming toward an M1-like phenotype

TME, tumor microenvironment; NK, natural killer; TAMs, tumor-associated macrophages.

## Potential area

Long and very long chain fatty acid uptake across the cell membrane is known to be facilitated by CD36, and it was recently demonstrated that very long chain fatty acid metabolism is critical for acute myeloid leukemia (AML) cell survival. As a result, CD36 represents a possible pharmaceutical target for AML fatty acid uptake suppression. Evidence that AML patients with high CD36 expression had a bad prognosis lends more credence to this (122). Also, the transporter of fatty acids, CD36, facilitates the metabolism of fatty acids and encourages the growth and spread of ovarian cancer (123). Since CD36 inhibition inhibits both tumor growth and M2 macrophage polarization, there may be two advantages to utilizing this transporter inhibitor to treat cancer (124). It is known that blocking important regulatory cytokines has therapeutic potential to improve anti-tumor immune activity because of their ability to enhance effector T-cell function, for example, IL-10 and TGF-β (**Table 3**). One potent, rapidly developing area of medicine is engineered cell-based therapeutics. Cells may carry out advanced activities like cell-mediated death, prolonged therapeutic synthesis *in situ*, and guided trafficking within the body, all of which are now beyond the scope of pharmaceutical techniques (125). The capacity of macrophages to migrate to the TME is very significant. Consequently, this drug’s delivery issue may be resolved by altering and adopting macrophages. In preclinical research and clinical trials, genetically engineered macrophages (GEMs), which are designed to deliver therapeutic payloads to trigger immune responses, have demonstrated promise (102).

**Table 3:** Therapeutic strategies targeting NK–macrophage crosstalk.

Strategy	Target	Metabolic effect	Impact on NK–macrophage cooperation
IL-15 agonists	NK activation	Increased glycolysis	Restores cytotoxic loop
IL-10 blockade	IL-10/IL-10R axis	Reduced immunosuppressive metabolism	Restores cooperation via macrophage reprogramming and enhanced antigen presentation
TGF-β blockade	TAM suppression	Reduced M2 skewing	Restoration of NK cell function
CD36 inhibition	Lipid uptake	Reduced FAO in TAMs	Rebalances crosstalk
Adenosine blockade	A2A	Reduced cAMP	Rescues NK cell function and supports macrophage–NK cooperation
STAT3 inhibitors	IL-10 axis	Reduced immunosuppression	Restores cooperation
mTOR modulation/inhibition	mTORC1 signaling	Reprograms cellular metabolism and limits immunosuppressive adaptation	Enhances NK persistence and normalizes macrophage metabolic polarization
GSK3β inhibition	GSK3β/β-catenin axis	Metabolic and transcriptional reprogramming	Rescues NK cell function and supports macrophage–NK cooperation

NK, natural killer; TAM, tumor-associated macrophage; FAO, fatty acid oxidation.

## Cell-to-cell interactions between NK cells and macrophages in the TME

### HLA-E-NKG2A/CD94

In the TME, leukocyte immunoglobulin-like receptors (LILRs), killer cell immunoglobulin-like receptors (KIRs), and the CD94/NKG2A heterodimer, NK cells largely engage with MHC I ligands on the macrophage surface. Under normal conditions, M1-type macrophages exhibit elevated Qa-1b expression, shielding them from NK-mediated cell death (126). Furthermore, NK cells’ IFN-γ release is further stimulated by the proinflammatory cytokines secreted by M1-type macrophages, such as IL-15, IFN-β, IL-18, and IL-12. M1–NK cell connection amplifies anti-cancer activity and creates a positive feedback loop. Nevertheless, HLA-E molecules are also expressed by M2-type macrophages. Ischemia and hypoxia in gliomas increase the expression of HLA-E on infiltrating macrophages and microglia. In addition to protecting TAMs from NK-dependent lysis, HLA-E-NKG2A/CD94 binding encourages NK cells to produce immune-suppressive cytokines, including TGF-β and IL-10 (100). NKG2A expression on the surface of NK cells can be further induced by TGF-β in the TME. The humanized IgG4 antibody *monalizumab* has a strong affinity and specificity for the NKG2A/CD94 inhibitory checkpoint receptor. Monalizumab’s NKG2A/CD94 binding disruption inhibits tumor-induced signaling on T and NK cells. Monalizumab is undergoing several clinical investigations. Crucially, monalizumab demonstrated additive effects that promoted both NKG2A+ PD-1+ NK and CD8+ T-cell effector functions when combined with durvalumab, an anti-PD-L1 blocking mAb. Additionally, monalizumab increased the NK cell-mediated antibody-dependent cellular cytotoxicity (ADCC) when combined with cetuximab, an anti-epidermal growth factor receptor (EGF-R) mAb that promotes ADCC. This suggests that investigating how monalizumab may enhance the positive effects of other oncology treatments would be worthwhile (127, 128).

### NKG2D-NKG2D ligand (NKG2DL)

NKG2D ligands are expressed by macrophages (NKG2DLs). In most cases, the NKG2DL–NKG2D interaction between NK cells and macrophages creates a positive feed-forward loop that increases the cytotoxicity and recruitment of NK cells while enhancing the anti-tumor activity of macrophages with M1-type polarization (65, 100). Similarly, during the *CD48–2B4 interaction*, after constitutively interacting with its high-affinity ligand 2B4 (also known as CD244 or SLAMF4) on NK cells, CD48 recruits the small adaptor protein SLAM-associated protein (SAP) to transduce costimulatory signals, which in turn causes NK-mediated cytotoxicity and the generation of IFN-γ. Complementing the role of the CD48–2B4 *C-type lectin-like receptor (CTLR) activation* axis provides another layer of regulation in NK cell function. Tumoricidal activity and effective NK cell function are intimately related to the CTLR family, a key pattern recognition receptor (PRR) for macrophages. Through the Dectin-1–IRF5–INAM pathway, macrophages in the TME identify N-glycan structures unique to tumor surfaces, hence increasing the effectiveness of NK cells in eliminating malignancies (129). Beyond the CTLR activation, the *CD155/CD122 interaction* further exemplifies the intricacies of NK cell-mediated immune activity. Poliovirus receptor (PVR) CD155 and poliovirus receptor-related family 2 (PVRL2) CD112, which are mostly found on macrophages, interact with DNAX accessory molecule-1 (DNAM-1/CD226), a stimulatory adhesion receptor, in both humans and mice to control the function of NK cells. The DNAM-1–CD155/CD112 interaction facilitates NK cell activation by promoting NK cell adherence to target cells, cytolytic activity, and IFN-γ production (65, 130). However, the *ICAM-1–LFA-1 interaction* plays a vital role in immune cell adhesion and signaling, facilitating effective communication between immune cells and their targets. Tumor cells and macrophages in the TME are the primary sources of expression for intercellular adhesion molecule-1 (ICAM-1, also known as CD54), a member of the immunoglobulin superfamily. According to reports, ICAM-1 expression in macrophages inhibits M2-type polarization and macrophage recruitment, preventing tumor dissemination in human colon cancers. NK cells carry a kind of integrin called LFA-1 (CD11a/CD18), which binds to ICAM-1 and facilitates NK cell motility and firm adherence at inflammatory areas. Cytokine-induced memory and maturation, NK cell activation, and cytotoxicity are all improved by the ICAM-1–LFA-1 relationship (65).

Macrophages can directly detect tumor-associated N-glycan complexes via the C-type lectin receptor Dectin-1, triggering a signaling cascade that activates IRF5. Transcription induced by IRF5 causes the expression of INAM (IRF5-dependent NK-activating molecule) on macrophages, which is essential for cell-to-cell contact-dependent NK cell activation. Macrophages augment NK cell cytotoxicity and IFN-γ production against tumor cells via INAM-mediated interactions (131). The Dectin-1–IRF5–INAM axis highlights the mechanism by which macrophage detection of tumor-specific glycans leads to efficient NK cell activation in the TME (132, 133).

## Spatial relations in TME

The spatial arrangement within the TME significantly influences the functional regulation of immune responses, as it mediates the impact of metabolic limitations. The proximity of cells facilitates direct interactions and paracrine signaling, which in turn modulate immune activation, suppression, and tissue remodeling processes. Consequently, in solid tumors, the effects of metabolic stressors, including hypoxia, nutrient deprivation, and lactate accumulation on immune cell positioning and functionality, are contingent upon distance-dependent interactions (134).

Increasing evidence indicates that spatial relationships between immune cells and tumor cells modulate immune efficacy, as ligand–receptor interactions and checkpoint engagement require close physical proximity. For example, the spatial distribution of immune targets such as PD-1 and PD-L1 varies across tumor niches and cell types, influencing local immune suppression and anti-tumor activity (135, 136).

Advancements in spatially resolved technologies, such as imaging techniques and ligand–receptor mapping, have facilitated in-depth investigations of cell–cell interactions within the TME. These methodologies elucidate the influence of spatial heterogeneity on the variability observed in immune responses and therapeutic results, thereby reinforcing the developing concepts of spatial biomarkers for predicting immunotherapy efficacy (137). Consequently, spatial organization constitutes a crucial dimension through which metabolic changes influence macrophage–NK cell interactions and the overall effectiveness of anti-tumor immunity.
